# Validation and Improvement of COCTS/HY-1C Sea Surface Temperature Products

**DOI:** 10.3390/s22103726

**Published:** 2022-05-13

**Authors:** Feizhou Zhang, Yulin Zhang, Zihan Zhang, Jing Ding

**Affiliations:** 1 Beijing Key Lab of Spatial Information Integration and 3S Application, Institute of Remote Sensing and Geographic Information System, School of Earth and Space Science, Peking University, Beijing 100871, China; zhangfz@pku.edu.cn (F.Z.); zhangyulin@stu.pku.edu.cn (Y.Z.); zzh_cytus@pku.edu.cn (Z.Z.); 2 Key Laboratory of Space Ocean Remote Sensing and Application, National Satellite Ocean Application Service, Ministry of Natural Resources, Beijing 100081, China

**Keywords:** COCTS/HY-1C, NEAR-GOOS, sea surface temperature, nonlinear regression, regularization

## Abstract

In oceanographic study, satellite-based sea surface temperature (SST) retrieval has always been the focus of researchers. This paper investigates several multi-channel SST retrieval algorithms for the thermal infrared band, and evaluates the accuracy of the COCTS/HY-1C SST products. NEAR-GOOS in situ SST data are utilized for validation and improvement, and a three-step matching procedure including geographic location screening, cloud masking, and homogeneity check is conducted to match in situ SST data with satellite SST data. Two improvement schemes, including nonlinear regression and regularization iteration, are proposed to improve the accuracy of the COCTS/HY-1C SST products and the typical application scenarios and the algorithm characteristics of these two schemes are discussed. The standard deviation of residual between retrieved SST and measured SST for these two data improvement algorithms, which are considered as the main indexes for assessment, result in an improvement of 13.245% and 14.096%, respectively. In addition, the generalization ability of the SST models under two data improvement methods is quantitatively compared, and the factors affecting the model accuracy are also carefully evaluated, including the in situ data acquisition method and measurement time (day/night). Finally, future works about SST retrieval with COCTS/HY-1C satellite data are summarized.

## 1. Introduction

The sea surface temperature (SST) reflects the thermophysical characteristics of the ocean surface, and it is one of the most widely used marine elements for researchers. It is of great significance in climate prediction, marine biology, marine chemistry, and marine geology. SST data are usually obtained by survey ship measurement, buoy measurement, or satellite remote sensing retrieval [1,2,3,4]. Two types of field observation techniques, namely the survey ship measurement and buoy measurement, can acquire high precision SST data. However, these two data acquisition techniques are limited by the measuring cost and the failure to meet the needs of real-time and wide-range monitoring [5]. The satellite remote sensing technology has a higher temporal resolution and spatial resolution, while data acquisition is more accessible and convenient, which makes up for the shortcomings of the two in situ measurement technologies [5,6].

At present, two types of satellite remote sensing technologies, namely the passive microwave remote sensing and infrared (middle infrared/thermal infrared) remote sensing, are mainly used for SST retrieval [7]. The basic principle is to receive the emitted radiation (microwave radiation/thermal radiation) from the ocean surface, and then calculate the SST using the Planck equation. In this paper experiment, band 9 (10.300–11.300 μm) and band 10 (11.500–12.500 μm) of Chinese Ocean Color and Temperature Scanner (COCTS) carried by the HY-1C satellite, are used to retrieve SST [8].

In the early studies on SST retrieval, only a single thermal infrared band is used in the algorithm [9]. The single band algorithm is based on the direct solution of the thermal radiation transfer equation. Therefore, the accuracy of the single-band algorithm is often limited due to the difficulty to obtain accurate atmospheric parameters [10]. One of the earliest multi-band algorithms is the Multi-Channel SST (MCSST) algorithm [4]. The MCSST algorithm proves that the combination of multi-band brightness temperature can efficiently reduce the impact of atmospheric absorption on SST retrieval, especially in areas having strong water vapor absorption. After the existence of the MCSST algorithm, the multi-band algorithms became the mainstream [11]. Multi-band algorithms with higher accuracy, such as the Cross-Product SST (CPSST) [12], Water Vapor SST (WVSST) [13], and Non Linear SST (NLSST) [14] algorithms, have been released since then and are widely used in the satellite SST products. The MCSST algorithm is the basis of the COCTS/HY-1C SST products, while the NLSST algorithm is the basis of the Advanced Very High-Resolution Radiometer (AVHRR), MODerate resolution Imaging Spectroradiometer (MODIS), and Visible Infrared Imaging Radiometer Suite (VIIRS) SST products [15,16,17]. This paper verifies the accuracy of the COCTS/HY-1C SST products.

To improve the COCTS/HY-1C SST products, two algorithms are proposed, namely enhanced Non-Linear SST (eNLSST) algorithm and regularized iteration SST (RISST) algorithm. Compared with the existing multi-band algorithms, the proposed two algorithms show a lower dependency on auxiliary data (reference SST data, water vapor data, cross validation product data, etc.), and only COCTS/HY-1C satellite level 1 and level 2 data are required for algorithm input. Experiments are conducted in this paper to prove that the performance of these two methods are remarkable and the best application scenarios for these two methods are discussed.

## 2. Data

### 2.1. Measured SST Data in NEAR-GOOS Database

The Northeast Asian Regional Global Ocean Observing System (NEAR-GOOS) is a regional experimental project of the Global Ocean Observing System (GOOS), which is operated by China, South Korea, Japan, and Russia. All the members participating in this cooperation can share and use the real-time monitoring data in the data management system, the ocean data in the Regional Delay Mode DataBase (RDMDB), the infrastructure in the marine monitoring system, and the communication network platform [18].

The RDMDB data of the NEAR-GOOS project are jointly implemented and published by Japan Oceanographic Data Center (JODC) and Japan Meteorological Agency (JMA). The measured data used in this paper experiment are taken from the SST decoded data in NEAR-GOOS RDMDB (https://near-goos1.jodc.go.jp/) (accessed on 31 March 2022).

**Figure 1:**
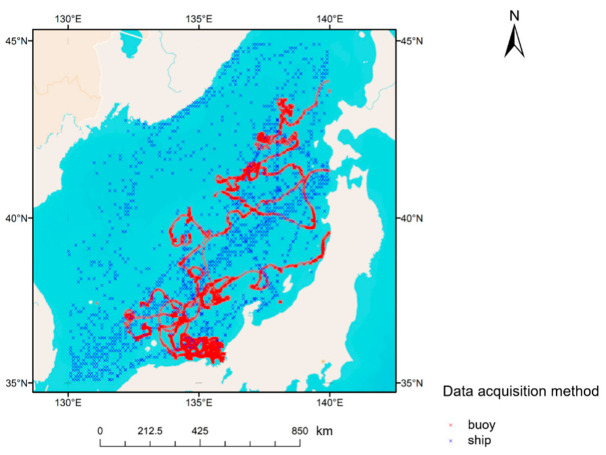
Space distribution of measured SST in NEAR-GOOS database.

In the experiment, the research area for SST retrieval is the subset of the Sea of Japan having a latitude of 35° N–45° N and a longitude of 130° E–140° E. The time span is from 1 January 2019 to 31 December 2019. The measured SST data are from the NEAR-GOOS database, including the data collected by survey ship and buoy. The spatial distribution of the measured data is shown in Figure 1, where the red “x” refers to the SST data obtained by buoy measurement, and the blue “x” denotes the SST data obtained by ship measurement. The amount of data obtained by buoy measurement is much larger than that obtained by survey ship measurement. The red trace represents the drift track of buoys, and the blue sparse trace represents the track of the survey ship.

Since previous research [13,14,15] and the algorithm for long-wave infrared SST products aboard MODIS and VIIRS (https://oceancolor.gsfc.nasa.gov/atbd/sst/) (accessed on 31 March 2022) indicate that satellite skin SST is in good agreement with buoy SST and ship SST. Therefore, the skin effect and diurnal heating effect are not taken into account in our experiment.

### 2.2. HY-1C Satellite Products

The HY-1C satellite was launched in September 2018. It has dramatically improved the observation accuracy and range, compared with HY-1A and HY-1B (https://osdds.nsoas.org.cn/HY1C_introduce) (accessed on 31 March 2022). In this paper, the thermal infrared data used for SST retrieval are from the COCTS sensor equipped on HY-1C. Table 1 presents the parameters of COCTS bands. The noise equivalent temperature difference (NEΔT) and temperature detection range of band 9 and band 10 meet the requirements of SST retrieval.

After geographic positioning, band registration, strip elimination, and radiometric correction, COCTS L1 data products are generated. They are also divided into L1A products and L1B products, according to whether an absolute radiation correction is conducted. L2 products are divided into three categories: (1) L2A products, that are basic products, including remote sensing reflectance of each band and aerosol-related parameters; (2) L2B products, that are standardized products, including chlorophyll concentration, total suspended solids concentration, suspended sediment concentration, 565 nm normalized water-leaving radiance, sea surface temperature, and water diffuse attenuation coefficient; (3) L2C products, that are experimental products and expanded products, including total pigment concentration, sun-glint coefficient, and water transparency.

The satellite data used in the SST retrieval experiment are the top-of-atmosphere (ToA) radiance L1B products of band 9 and band 10, remote sensing reflectance L2A products data of band 8 and L2B SST products.

## 3. Experimental Methods

### 3.1. Establishing Blackbody Radiation Lookup Table in Thermal Infrared Band of HY-1C

Since the brightness temperature is the independent variable of the SST retrieval model using the thermal infrared multi-band algorithm, it is necessary to establish the blackbody radiation lookup table of COCTS according to the band response functions of band 9 and band 10. The quantitative relationship between ToA radiance and brightness temperature is expressed as:

(1) $$L_{i}=B(T_{i})=\sum f_{i}(\lambda )\times \frac{2hc^{2}}{\lambda^{5}}\times \frac{1}{e^{hc/\lambda KT_{i}}-1}$$

where *i* represents the band number, *B*(*T_i_*) is the blackbody radiation (denoted by *L_i_*), *T_i_* denotes the temperature, *f_i_*(*λ*) represents the band response function, *λ* is the wavelength, *c* denotes the velocity of light, *h* represents the Planck constant, and *K* is the Boltzmann constant.

In the range of 200–300 K, *T_i_* is set with an interval of 0.001 K in order to calculate the blackbody radiation *B*(*T_i_*). In the calculation process, the Planck constant is *h* = 6.626 × 10^−34^ J⋅s, the Boltzmann constant is *K* = 1.381 × 10^−23^ J/K, the unit of wavelength *λ* is m, the unit of *T_i_* is K, and the unit of calculation result (blackbody radiation *B*(*T_i_*)) is 10^−6^ W/(m^2^⋅sr⋅μm).

After the blackbody radiation look-up tables of band 9 and band 10 are established, the ToA radiance of the thermal infrared band of L1B products is converted into brightness temperature using the blackbody radiation look-up table and linear interpolation:

(2) $$T_{i}=T_{m}+(T_{n}-T_{m})\times \frac{B(T_{i})-L_{m}}{L_{n}-L_{m}}$$

where *T_i_* is the brightness temperature after conversion, *B*(*T_i_*) is the ToA radiance of L1B products, *L_m_* and *L_n_* are the radiance values in the blackbody radiation lookup table that meet *L_m_* < *B*(*T_i_*) < *L_n_*, and *T_m_* and *L_m_* are the lookup results of *L_m_* and *L_n_*, respectively.

### 3.2. Data Matching

For the verification and improvement of HY-1C SST products, it is necessary to match the satellite data with the measured data.

**Figure 2:**
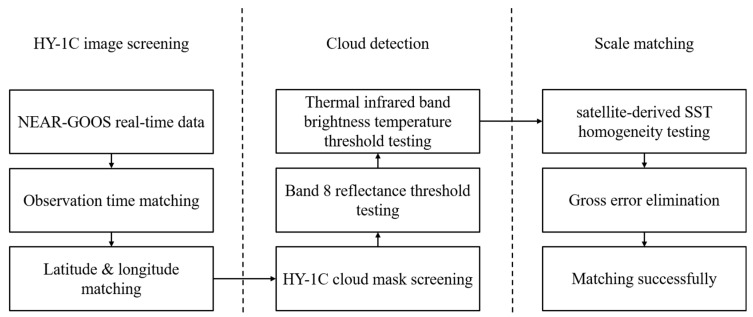
Data matching process between HY-1C satellite data and NEAR-GOOS real-time data.

Technically speaking, the measured SST reflects the subsurface temperature at a certain depth and the satellite-retrieved SST reflects the bulk temperature (https://www.ghrsst.org/wp-content/uploads/2021/04/SSTDefinitionsDiscussion.pdf) (accessed on 31 March 2022) influenced by the variability of the whole area (for HY-1C, one pixel refers to 1.1 km × 1.1 km area) and the contribution from the near-surface temperature gradients. Since the measured in situ SST should well represent the satellite-retrieved SST, data matching is needed. To eliminate the influence of the scale effect, a regional homogeneity test is applied during the data matching process. The data matching process can be divided into three steps: (1) preliminary screening of HY-1C satellite images; (2) cloud detection; and (3) scale matching. The data matching process is illustrated in Figure 2.

The HY-1C satellite image is first preliminarily screened through the observation time, longitude, and latitude, in order to preliminarily select candidate satellite images that have the possibility of matching with real-time NEAR-GOOS measured data.

Cloud detection is then performed to remove cloudy pixels. After using the HY-1C cloud mask, further judgment is made according to the remote sensing reflectance of band 8 and the brightness temperature of the thermal infrared bands. In general, the ToA reflectance is higher than the sea surface reflectance. In addition, the ToA brightness temperature is lower than the sea surface brightness temperature. Therefore, when the remote sensing reflectance of band 8 is higher than 0.25 or the brightness temperature of the thermal infrared bands is less than 265 K, the pixel is judged to be covered by clouds.

Afterwards, the scale difference is taken into consideration. The survey ship measurement data and buoy measurement data in the NEAR-GOOS dataset are both point measurement data. However, the spatial resolution of the HY-1C is 1.1 km, which indicates that the satellite SST pixel data refer to the comprehensive contribution of the pixel coverage area. When the SST of the 1.1 km × 1.1 km area is relatively homogeneous, the NEAR-GOOS point measurement data can well represent the satellite-retrieved SST. The homogeneity is determined by the standard deviation of a 5 × 5 pixel-window with the target SST pixel at the center. When the standard deviation is less than 0.500 K, it is considered that the stability of pixel SST is appropriate, and therefore this pixel SST data can match with the NEAR-GOOS in situ point data.

Finally, the gross error is eliminated. When the absolute error between HY-1C satellite SST data and NEAR-GOOS measured SST data exceeds 2 K, it is considered as gross error for elimination. After these steps are completed, the HY-1C SST data and NEAR-GOOS measured SST data are successfully matched, with a number of successfully matching pairs equal to 896 (each pair refers to a grid point with a resolution of 1.1 km × 1.1 km).

### 3.3. Improvement for HY-1C SST Products

The SST product algorithm of HY-1C satellite is based on the MCSST algorithm, which basic equation is given by [4]:

(3) $$T_{MCSST}=C_{1}T_{i}+C_{2}(T_{i}-T_{j})+C_{3}$$

where *T_MCSST_* is the predicted SST, *T_i_* is the brightness temperature of band *i*, *T_j_* is the brightness temperature of band *j*, and *C*_1_, *C*_2_, and *C*_3_ are the algorithm coefficients.

The SST product algorithm of the AVHRR, MODIS, and VIIRS sensors is based on the NLSST algorithm, which basic equation is given by [15]:

(4) $$T_{NLSST}=C_{1}T_{i}+C_{2}(T_{i}-T_{j})T_{ref}+C_{3}(\sec\theta -1)(T_{i}-T_{j})+C_{4}$$

where *T_NLSST_* is the predicted SST, *T_ref_* is the reference SST, *T_i_* is the brightness temperature of band *i*, *T_j_* is the brightness temperature of band *j*, *θ* is the observation zenith angle, *C*_1_, *C*_2_, *C*_3_, and *C*_4_ are the algorithm coefficients.

Based on the reference SST *T_ref_*, the NLSST algorithm used by AVHRR, MODIS, and VIIRS eliminates the influence of the atmospheric effect by linear combination of brightness temperature difference. Considering MODIS as example, the document of MODIS SST products (https://oceancolor.gsfc.nasa.gov/atbd/sst/) (accessed on 31 March 2022) indicates that the reference SST *T_ref_* comes from AVHRR SST products [2,3]. After further eliminating the influence of the atmospheric absorption effect using the NLSST algorithm, the standard deviation of the residual between the measured temperature and predicted temperature for MODIS/Terra is 0.510 K. In addition, the standard deviation of the residual between the measured temperature and predicted temperature for MODIS/Aqua is 0.509 K.

The MCSST algorithm used by the HY-1C satellite is more concise than the NLSST algorithm. In this study, two schemes are used to improve the SST products:

The first scheme considers the MCSST retrieval results as the reference SST data, and applies the NLSST algorithm in order to increase the accuracy, namely enhanced NLSST (eNLSST) algorithm:

(5) $$T_{improved}=C_{1}T_{i}+C_{2}(T_{i}-T_{j})T_{MCSST}+C_{3}(\sec\theta -1)(T_{i}-T_{j})+C_{4}$$

where *T_improved_* is the predicted SST, *T_ref_* is the reference SST, *T_i_* is the brightness temperature of band *i*, *T_j_* is the brightness temperature of band *j*, *θ* is the observation zenith angle, and *C*_1_, *C*_2_, *C*_3_, and *C*_4_ are the algorithm coefficients.

The second scheme is originally proposed in this paper. It is referred to as regularized iteration SST (RISST) algorithm, which is based on regularization iteration. In the RISST algorithm, the *C*_3_ constant in the MCSST algorithm is replaced by the Euclidean norm (L2 norm) regularization term. The HY-1C products is considered as a starting point, and a nonlinear model is built through iterations. Adding the regularization term helps to control the degree of overfitting, and improves the retrieval accuracy. The basic equation of the RISST algorithm is given by:

(6) $$T_{k}-T_{k-1}=C_{k1}T_{i}+C_{k2}(T_{i}-T_{j})+\lambda (C_{k1}^{2}+C_{k2}^{2})$$

where *T_k_* is the SST calculated in iteration *k*, *T_k_*_−1_ is the SST calculated in iteration *k* − 1, *T_i_* is the brightness temperature of band *i*, *T_j_* is the brightness temperature of band *j*, *C_k_*_1_ and *C_k_*_2_ are the algorithm coefficients of iteration *k*, *λ*(*C_k_*_1_^2^ + *C_k_*_2_^2^) is the L2 norm regularization term, and *λ* is the regularization term coefficient which is determined by *C*_1_, *C*_2_, and *C*_3_ fitted by the MCSST algorithm.

The RISST algorithm uses the iterative method, which is rarely used in the existing SST retrieval algorithm. Moreover, the regularization term correction is added to solve the overfitting problem of the iterative method.

Both eNLSST algorithm and RISST algorithm are tested in our experiment to examine their difference in performance, and the advantages and disadvantages of these two data improvement methods are analyzed in later sections.

### 3.4. Accuracy Evaluation Method

According to the NASA SST products evaluation standard [15], the accuracy verification of SST retrieval is mainly performed by analyzing the mean, median, standard deviation, and robust standard deviation of the residual between the predicted SST and measured SST [19,20,21,22,23]. The standard deviation and robust standard deviation can be calculated as:

(7) $$\mathrm{SD}=\sqrt{\frac{\sum res}{n}}$$

(8) $$\mathrm{RSD}\approx \frac{res_{0.75}-res_{0.25}}{k}$$

where SD is the standard deviation (SD), RSD is the robust standard deviation (RSD), *res* is the residual between predicted SST and measured SST, *res*_0.75_ is the upper quartile of the residual between predicted SST and measured SST, *res*_0.25_ is the lower quartile of the residual between predicted SST and measured SST, *k* is the coefficient of robust standard deviation (Note that the robust standard deviation helps to avoid the problems of heteroscedasticity and self-correlation, and the value of *k* can be calculated through software Stata).

As pointed out in Section 2.1, the skin effect and diurnal heating effect are not taken into account in our experiment. This would lead to systematic error which influences the reliability of mean and median values, but this bias value would not influence SD and RSD since SD and RSD represent the statistical dispersion of the residual between predicted SST and measured SST. Therefore, SD and RSD are taken as the candidate indicators for algorithm comparison.

## 4. Results

### 4.1. Data Matching Results and Analysis

**Figure 3:**
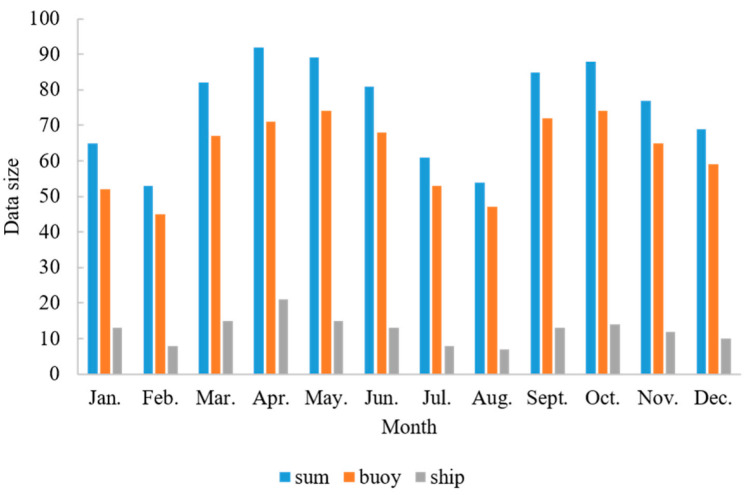
Data matching results between HY-1C image data and NEAR-GOOS real-time data.

After three main steps (preliminary screening, cloud detection and scale matching), the NEAR-GOOS measured data are successfully matched with the HY-1C satellite data, and 896 pairs are generated. Among all 896 matching pairs, 747 pairs are matched between HY-1C satellite data and buoy measurement data, and 149 pairs are matched between HY-1C satellite data and survey ship measurement data. The monthly distribution of matching data is shown in Figure 3. It can be seen from that: (1) The amount of successfully matched buoy survey data is significantly larger than that of the ship survey data in each month, and the ship survey data account for 15.094–29.578% in the total data. (2) There is a fluctuation in the amount of matched data each month, which is due to the cloud coverage and data quality fluctuation. During the period from January 2019 to February 2019, many data are eliminated due to cloud coverage, and during the period from July 2019 to August 2019, many data are eliminated because of their poor quality. (3) Due to the limited number of matching points, three months are considered in this paper as the interval for data division, in order to ensure that each group has 200–300 matching points for the SST retrieval model training.

### 4.2. SST Retrieval Results and Analysis

The three SST retrieval algorithms are compared: MCSST algorithm (algorithm of generating COCTS/HY-1C SST products), eNLSST algorithm (the first improvement scheme, which considers the COCTS/HY-1C SST products as the reference SST to conduct the eNLSST algorithm), and RISST algorithm (the second improvement scheme, which applies the regularization iteration scheme to acquire higher accuracy).

These SST retrieval algorithms are divided by latitude zone (the latitude zone interval is 5°), day and night, and time (the time interval is three months). Seventy percent of the matching points are used to fit the model parameters and the remaining 30% of the matching points are used to do the accuracy assessment. This process is repeated 10 times in order to reduce the accidental error. The comparison results are shown in Table 2.

It can be seen from Table 2 that: (1) in this paper, the two data improvement algorithms (eNLSST and RISST algorithms) perform better than the product algorithm. Considering SD as the main index, the improvement range of the eNLSST and RISST algorithms is 13.245% and 14.096%, respectively. This proves the feasibility of the proposed schemes. (2) The overall accuracy of the RISST algorithm is slightly better than that of the eNLSST algorithm. However, the RISST algorithm is more time-consuming than the eNLSST algorithm. This is due to the fact that the RISST algorithm applies a regularization iteration scheme which leads to higher time complexity. (3) Compared with the eNLSST algorithm and RISST algorithm, the mean and median of the MCSST algorithm are closer to 0, which indicates that the residual distribution between the retrieval result of the MCSST algorithm and the measured value are more symmetrical. However, the absolute value of the residual is larger, which indicates that more fluctuation of the retrieval results exists. The retrieval results of the eNLSST algorithm and RISST algorithm tend to be the opposite to the MCSST algorithm, which is less symmetrical but more stable.

## 5. Discussion

### 5.1. Research on Model Generalization Ability

In order to study the generalization ability of the SST retrieval model developed by the eNLSST and RISST algorithms, two data division methods are used: (1) OF1: 70% matching points are randomly chosen as the training set, and the remaining 30% matching points are considered as validation set. (2) OF2: 70% of the matching points are randomly chosen as training set, and the training set is also used for validation. These two-division methods are repeated 10 times. The performance of the eNLSST and RISST algorithms with group OF1 and OF2 is shown in Table 3.

It can be seen from Table 3 that: (1) By considering SD as the main index, and comparing the results of the eNLSST and RISST algorithms with the division of OF1 and OF2, respectively, it can be deduced that the SD of OF2 is lower than that of OF1. This indicates that the dispersion degree of OF2 is lower than that of OF1, which demonstrates that both algorithms have an overfitting problem to some extent. (2) The overfitting problem of the RISST algorithm is more severe than that of the eNLSST algorithm. However, it is still acceptable due to the regularization iteration scheme, which indicates that the eNLSST algorithm has a better generalization ability, and the generalization ability of the RISST algorithm is weaker but still meets the application requirement. In further study, it is discovered that iterations of the RISST algorithm are under 3 in most cases, which leads to a less severe problem of overfitting. If the iteration is beyond 3 in most cases, the overfitting problem will be too severe to apply in product generation.

**Figure 4:**
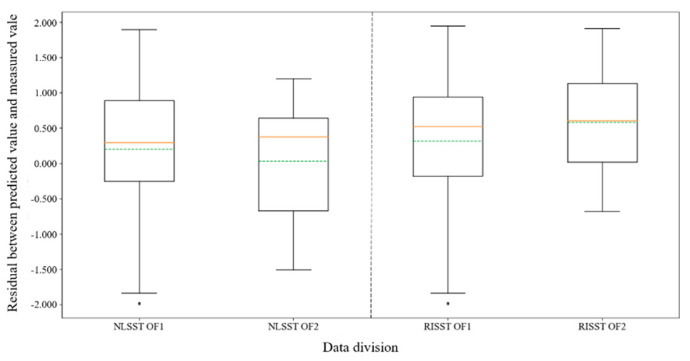
Residual boxplot of the eNLSST and RISST algorithms with group OF1 and OF2.

The boxplot of residual distribution with group OF1 and OF2 is shown in Figure 4. The boxplot shows the mean value, minimum value, lower quartile value, median value, upper quartile value, and maximum value of the residual between predicted SST and measured SST. The green dotted line in the boxplot represents the mean value, while the solid orange line represents the median value. It can be seen from the boxplot that the dispersion degree of residual distribution with group OF2 is lower than that of OF1 (i.e., the *y*-axis span for OF2 boxplot is smaller).

### 5.2. Analysis concerning Data Acquisition Method and Research Time

The measured data can be divided into two groups: survey ship data and buoy data. In order to evaluate the influence of the acquisition method of measured data on the SST retrieval accuracy, the data are divided into two groups: (1) B1: only buoy data are used, and the validation method is similar to OF1; (2) B2: only survey ship data are used, and the validation method is similar to OF1. The performance of the eNLSST and RISST algorithms with group B1 and B2 is shown in Table 4.

It can be seen from Table 4 that: (1) For both eNLSST and RISST algorithms, the accuracy of group B2 trained with survey ship measured data is lower than that of group B1 trained with buoy measured data. (2) The eNLSST algorithm has a weak ability to resist the systematic error and gross error of the trained data, while the RISST algorithm relies less on the original data quality, and has a better ability to resist error. (3) By comparing group B1 and B2 with group OF1 which mixes up survey ship data and buoy data for training, it can be deduced that there is only slight influence of using mixture data for retrieval (the survey ship data account for 15.094–29.578%).

In order to increase the amount of training data, mixture data are used in the proposed main models, regardless of the data acquisition methods. Nevertheless, it is suggested to use buoy data for training and validation. More precisely, the survey ship SST data are measured after the sea surface water is drawn into the cabin. The process of water compression and pumping will affect the SST measurement accuracy, which leads to the fact that the survey ship measured SST is less accurate than the buoy measured SST.

The retrieval time (day/night) will also affect the accuracy of SST retrieval. In order to analyze the influence of the retrieval time (day/night) on the SST retrieval accuracy, the data are divided into two groups: (1) T1: only day-time SST data are considered for training and validation according to the same scheme as OF1; (2) T2: only night-time SST data are considered for training and validation according to the same scheme as OF1. The performance of the eNLSST and RISST algorithms with group T1 and T2 is shown in Table 5.

It can be seen from Table 5 that: (1) there is a significant difference between the day and night retrieval model for both the eNLSST and RISST algorithms, which indicates that it is necessary to separately fit the model coefficients according to day and night. (2) Theoretically, the SST retrieval accuracy at night-time should be higher than that during day-time, due to the influence of reflected solar radiation. More precisely, although the solar energy in the thermal infrared band is deficient, the water leaving radiance is also relatively low, making the reflected solar radiation energy still need to be considered. However, there is no apparent regularity in the relationship between day and night accuracy in the eNLSST and RISST algorithms. It is speculated that both algorithms use the linear/nonlinear combination of brightness temperature, which efficiently weakens the influence of the atmospheric absorption and off counteracts the influence of the reflected solar radiation energy to a certain extent.

**Figure 5:**
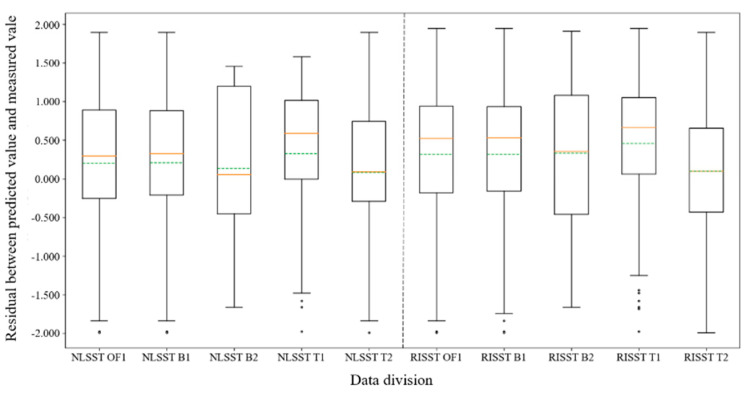
Residual boxplot of the eNLSST and RISST algorithms with group OF1, B1, B2, T1, and T2.

The boxplot of residual distribution with group OF1, B1, B2, T1, and T2 is shown in Figure 5. The boxplot intuitively supports the results of table analysis: (1) by comparing the results of B1 and B2 for the eNLSST and RISST algorithms, it is deduced that the gap between the maximum and minimum values under group B1 is larger than that of group B2. However, the gap between the upper and lower quartiles is smaller than that of group B2, which indicates that the residual distribution of group B1 is closer to the normal distribution, and the residual aggregation degree of group B1 is higher than that of group B2. (2) By comparing the results of OF1 and B1 for the eNLSST and RISST algorithms, it is deduced that the difference in residual distribution is slight, which indicates that although the survey ship data have higher accuracy, they slightly affect the overall retrieval accuracy due to the lack of survey ship data quantity. (3) By comparing the results of T1 and T2 for the eNLSST and RISST algorithms, it can be seen that there is a significant difference in the residual distribution between group T1 and group T2, judging from the maximum and minimum values and the difference between the upper and lower quartiles shown in the boxplot. This indicates that it is necessary to fit the model parameters according to the retrieval time (day/night), while there is no apparent regularity for day and night accuracy.

### 5.3. Limitations and Future Work

Firstly, although the overfitting problem of the RISST algorithm is acceptable for several applications, it is still more severe than that of the eNLSST algorithm. It is difficult to predict whether the generalization ability of the RISST algorithm will decrease as the training size increase. In future work, a comprehensive data division strategy should be developed to ensure the regional generalization ability of the model fitted by the RISST algorithm.

In addition, the time interval used in the experiment is three months. When the matching points are sufficient, one month can be considered as the time interval, which is supposed to obtain higher accuracy. Some previous works [15,16] have proved that using the data in the adjacent five months of the last year same period for weighted training can obtain higher accuracy (for example, using the data from January 2018 to May 2018 for training and applying different weights for each month to fit the model parameters of March 2019 SST retrieval. Since HY-1C started to provide satellite data service in September. 2018, the historical data are not sufficient for fitting the 2019 SST retrieval parameters. As the HY-1C satellite data accumulate, the weighted training method can be applied to improve the SST retrieval accuracy.

Moreover, although NASA uses the mean, median, SD, and RSD to assess the performance of SST retrieval model, this has some shortcomings, as this validation method does not directly evaluate the advantages and disadvantages of predicted SST, but the statistical distribution of residual between measured SST and predicted SST. For example, when the SD is low, it only indicates that using an offset value to correct the SST retrieval model will have a positive effect, which cannot show that the model is more accurate because the residual may gather in an interval with larger error and higher degree of aggregation. Furthermore, the current validation system includes four indicators. Therefore, it is difficult to comprehensively evaluate the model performance because only one indicator is considered as the main index to quantitatively assess the improvement of model performance (in this paper experiment, the SD is considered as the main indicator).

Finally, since this paper experiment is limited by computer storage space and computing power, the validation and improvement of SST products are only conducted in the Sea of Japan. In future work, when better hardware conditions are achieved and better multi-sensor data processing methods are developed [24], the research area could be expanded to a broader area and extended period.

## 6. Conclusions

In the Sea of Japan, the validation result of COCTS/HY-1C SST products is [mean: 0.019, median: −0.145, SD: 1.057, RSD: 1.158] (noted that this result is generated without removing the gross error). It is speculated that the accuracy of COCTS/HY-1C SST products may be affected by the inaccurate radiometric calibration or insufficient training data.

In this paper, a nonlinear regression and a regularization iteration strategy are used to improve the COCTS/HY-1C SST products. The eNLSST algorithm used in this paper considers the COCTS/HY-1C SST products as the reference SST. Taking SD as the main validation indicator, the accuracy of the eNLSST algorithm is improved by 13.245%, compared with the original SST products, that are generated by the MCSST algorithm. The proposed RISST algorithm is an iterative SST retrieval algorithm. It improves the model performance using an iteration process, and maintains the generalization ability by regularization correction. The MCSST algorithm parameters are used as the initial parameters of the iteration. Considering SD as the main validation indicator, the accuracy of the RISST algorithm is improved by 14.096% compared with the original SST products.

In the discussion, it is proved that the best application scenarios for these two data improvement methods are different. The RISST algorithm can alleviate the overfitting problem to a certain extent, which meets the needs of application, while its generalization ability is lower than that of the eNLSST algorithm. When extra-high accuracy is required for small scale study, RISST algorithm would perform better with higher accuracy. However, when it comes to large scale study and the training process are expected to be efficient, the eNLSST algorithm would outperform the RISST algorithm. The influence of the acquisition method of measured data and retrieval time (day/night) on the model performance is also discussed. It can be deduced that the accuracy of the survey ship data is lower than that of the buoy data, and the retrieval time (day/night) significantly affects the overall accuracy. Therefore, it is suggested to use buoy data for training and validation, and separately fit the model parameters for day-time and night-time.

As the satellite data of COCTS/HY-1C accumulate and more accurate radiometric calibration methods are applied, the SST retrieval of COCTS/HY-1C will be spatially and temporally expended to a wider range.

## Figures and Tables

**Table 1:** COCTS/HY-1C band parameters.

Band Number	Band /μm	Measurement Condition /mW·cm^−2^·sr^−1^·μm^−1^	Signal to Noise Ratio /SNR	Maximum Radiance /mW·cm^−2^·sr^−1^·μm^−1^
1	0.402–0.422	9.100	349	13.940
2	0.433–0.453	8.410	472	14.490
3	0.480–0.500	6.560	467	14.590
4	0.510–0.530	5.460	448	13.860
5	0.555–0.575	4.570	417	13.890
6	0.660–0.680	2.460	309	11.950
7	0.730–0.770	1.610	319	9.720/5.000
8	0.845–0.885	1.090	327	6.930/3.500
9	10.300–11.300	0.200 K (300 K, NEΔT)		320 K (Maximum brightness temperature)
10	11.500–12.500	0.200 K (300 K, NEΔT)		320 K (Maximum brightness temperature)

**Table 2:** Performance of the MCSST, eNLSST, and RISST algorithms (* denotes the proposed improvement algorithm).

Algorithm	Mean	Median	SD	RSD
MCSST	0.019	−0.145	1.057	1.158
eNLSST	0.206	0.300	0.917	0.824
RISST *	0.321	0.525	0.908	0.810

**Table 3:** Performance of the eNLSST and RISST algorithms with group OF1 and OF2.

Group	eNLSST				RISST			
	Mean	Median	SD	RSD	Mean	Median	SD	RSD
OF1	0.206	0.300	0.917	0.824	0.321	0.525	0.908	0.810
OF2	0.031	0.380	0.884	0.945	0.587	0.605	0.857	0.804

**Table 4:** Performance of the eNLSST and RISST algorithms with group B1 and B2.

Group	eNLSST				RISST			
	Mean	Median	SD	RSD	Mean	Median	SD	RSD
OF1	0.206	0.300	0.917	0.824	0.321	0.525	0.908	0.810
B1	0.213	0.325	0.904	0.790	0.319	0.530	0.904	0.794
B2	0.136	0.055	1.035	1.189	0.332	0.360	0.933	1.111

**Table 5:** Performance of the eNLSST and RISST algorithms with group T1 and T2.

Group	eNLSST				RISST			
	Mean	Median	SD	RSD	Mean	Median	SD	RSD
T1	0.089	0.090	0.895	0.747	0.096	0.100	0.947	0.786
T2	0.327	0.590	0.924	0.736	0.456	0.665	0.856	0.718

## Data Availability

HY-1C data (https://osdds.nsoas.org.cn/) (accessed on 31 March 2022) and NEAR-GOOS data (http://near-goos.nmdis.org.cn/catalog/home) (accessed on 31 March 2022).
